# Both Direct and Vicarious Experiences of Nature Affect Children’s Willingness to Conserve Biodiversity

**DOI:** 10.3390/ijerph13060529

**Published:** 2016-05-25

**Authors:** Masashi Soga, Kevin J. Gaston, Yuichi Yamaura, Kiyo Kurisu, Keisuke Hanaki

**Affiliations:** 1 Department of Urban Engineering, School of Engineering, The University of Tokyo, 7-3-1, Hongo, Bunkyo, Tokyo 113-8656, Japan; kiyo@env.t.u-tokyo.ac.jp (K.K.); hanaki@env.t.u-tokyo.ac.jp (K.H.); 2 Environment and Sustainability Institute, University of Exeter, Penryn, Cornwall TR10 9FE, UK; k.j.gaston@exeter.ac.uk; 3 Forestry and Forest Product Research Institute, Matsunosato 1, Tsukuba 305-8687, Japan; yamaura@ffpri.affrc.go.jp

**Keywords:** biodiversity conservation, biophilia, conservation psychology, ecosystem services, environmental education, global change, public health, human-nature interactions, pro-environmental behavior, well-being

## Abstract

Children are becoming less likely to have direct contact with nature. This ongoing loss of human interactions with nature, the extinction of experience, is viewed as one of the most fundamental obstacles to addressing global environmental challenges. However, the consequences for biodiversity conservation have been examined very little. Here, we conducted a questionnaire survey of elementary schoolchildren and investigated effects of the frequency of direct (participating in nature-based activities) and vicarious experiences of nature (reading books or watching TV programs about nature and talking about nature with parents or friends) on their affective attitudes (individuals’ emotional feelings) toward and willingness to conserve biodiversity. A total of 397 children participated in the surveys in Tokyo. Children’s affective attitudes and willingness to conserve biodiversity were positively associated with the frequency of both direct and vicarious experiences of nature. Path analysis showed that effects of direct and vicarious experiences on children’s willingness to conserve biodiversity were mediated by their affective attitudes. This study demonstrates that children who frequently experience nature are likely to develop greater emotional affinity to and support for protecting biodiversity. We suggest that children should be encouraged to experience nature and be provided with various types of these experiences.

## 1. Introduction

Children are spending less and less time outdoors interacting with nature (natural environments and associated biodiversity) than ever before [1,2,3], as we become an increasingly urban species [4,5]. In urban environments, a high proportion of the area is comprised of artificial materials and is segregated from natural systems and processes, which can result in a marked reduction in opportunities for interaction with nature [6,7]. Indeed, recent urban expansion and densification have both reduced the availability of neighborhood natural places [8,9]. Besides rapid urbanization, the dramatic growth in sedentary pastimes like watching television, playing computer games, and exploring the Internet has also decreased children’s time available for engaging in such interaction; the majority of children, especially in industrial countries, today spend the majority of their leisure time in screen-based entertainment [2]. Furthermore, other social factors, such as parental concerns for safety and academic pressures, also hinder children from exploring nature freely [10,11,12]. As a result, today’s children are highly disconnected from nature; the progressive loss of human interactions with nature is termed the “extinction of experience” [3,6,13].

Children’s daily contact with nature is viewed as an essential component in forging and reinforcing favorable feelings toward nature [13,14,15]. Indeed, it has repeatedly been demonstrated that the frequency of involvement with nature at an early age enhances individuals’ emotional bonds with, interest in, and care for nature [16,17,18,19]. In the U.S., Bixler *et al.* [16], for example, demonstrated that people reporting having frequently played in wild environments had more favorable perceptions of natural environments. In the U.K., Hinds and Sparks [20] also reported that children who had grown up in rural areas reported more positive orientation toward engaging with nature than those from urban areas, suggesting that childhood nature experiences have a long-lasting impact on individuals’ emotional connectedness to nature. As such, researchers and educators have believed that interaction with neighborhood natural environments is key to enhancing children’s positive feelings toward nature [15,17,19].

This said, as noted by Kellert [14], today’s children can experience nature in a wide variety of ways, which are not simply limited to involvement with “direct” experiences of nature (participating in nature-based activities), but includes “vicarious” nature experiences (*i.e.*, nature experiences without any actual, physical contact with natural settings, such as through magazines, books, films, television programs, and websites). Indeed, in recent years, there is an increasing number, and diversity, of opportunities for people to contact with digitally manipulated images of wildlife and natural scenes [21], and several studies suggest that these vicarious experiences of nature can have a significant impact on children’s attitudes toward it [22,23]. Nevertheless, how vicarious experiences are associated with individuals’ favorable feelings toward nature is still poorly understood.

A growing body of research has shown that individuals’ affective attitudes (a psychological tendency to respond positively or negatively toward a certain object) regarding nature play a key role in the decision-making process regarding biodiversity conservation and wildlife management [24,25,26,27]. In Kenya, for example, de Pinho *et al.* [27] observed that perceiving a species as beautiful or ugly was the primary factor explaining people’s support for its protection in or removal from their locale. Likewise, in China, Zhang *et al.* [19] found a significant positive influence of children’s favorable feelings toward neighborhood wildlife on their willingness to conserve it. A questionnaire survey of more than 600 Spanish citizens also revealed a strong correlation between people’s affective attitudes toward biodiversity and their willingness to allocate funds for conservation [25]. Bearing in mind these existing studies, loss of direct, and even vicarious, experiences of nature is likely to decrease children’s willingness to conserve biodiversity through the medium of reduced positive feelings (affective attitudes) toward it. However, quantitative studies assessing how children’s extinction of experience of nature affects their affective attitudes toward and willingness to conserve biodiversity remain scarce.

**Figure 1:**
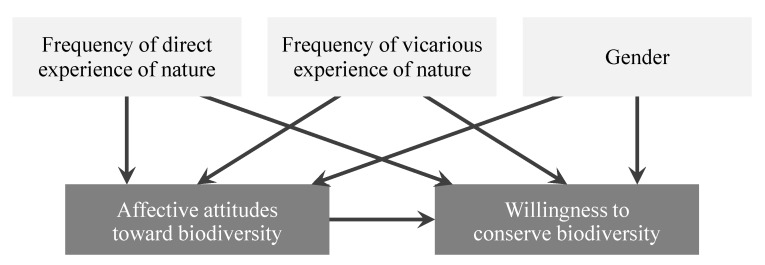
Hypothesized framework illustrating relationships between children’s experience of nature and their affective attitudes toward and willingness to conserve biodiversity. In this study, affective attitude toward biodiversity was used as a mediating factor between experience of nature and willingness to conserve biodiversity. Gender was included to control for its potential confounding effects on children’s attitudes toward and willingness to conserve biodiversity.

Understanding and addressing the role of both direct and vicarious experiences of nature in fostering children’s affective attitudes toward and willingness to conserve biodiversity is vital if we are to develop effective strategies and programs aimed at promoting public environmental awareness and action. Here, we conducted a questionnaire survey of elementary schoolchildren in Tokyo, Japan, and investigated the effects of frequency of direct (participating in nature-based activities in neighborhood natural environments) and vicarious experiences of nature (reading books or watching TV programs about nature and talking about nature with parents or friends) on their affective attitudes toward and willingness to conserve biodiversity. In doing so, this study focused on locally common animal species that children can encounter on a daily basis, rather than endangered or exotic species, and measured children’s conservation attitudes toward local biodiversity (for a similar approach, see [19]). As described above, we hypothesized that (1) direct and vicarious experiences with nature affect children’s affective attitudes toward and willingness to conserve biodiversity, and (2) children’s affective attitudes toward biodiversity act as a mediating factor between experience of nature and willingness to conserve biodiversity (Figure 1). We also explored gender differences in children’s attitudes toward animal species, as this is known to be a factor that can affect preferences for wildlife [19,28].

## 2. Experimental Design

### 2.1. Participants and Questionnaires

The surveys were conducted at an elementary school in Fuchu city, a western suburb of Tokyo metropolis, Japan, in May 2015. Fuchu city covers 29.43 km^2^ and has an estimated population of 256,716 residents [29]. At 25.4%, the greenspace coverage (e.g., parks and woodlands) in this city is comparable to that of the average of other regions of Tokyo, as is the socio-economic composition of its residents [29]. The study school was located next to the Tama River, the biggest river in Tokyo (Figure S1), and schoolchildren lived within the same school catchment area (approximately 1 km^2^ around the school). In the surrounding area of the school, although a large proportion was already developed, various types of natural environments where children could experience nature freely still remained, including urban parks, shrine groves, woodlands, grasslands, and riparian greenspace.

All children from grades three through six participated in the survey, a total of 397 (female = 202, male = 195), aged 9–12 years old (just excluding those who were absent on the day of the survey). The children participating in the survey were all made clearly aware of its purpose. The questionnaires were produced and administered jointly with, and approved by, the school’s principal and teachers. Because this study used data with no identifiable information on the survey children, neither formal ethics approval nor written consents from their parents were required.

Questionnaires were conducted with every class (taking approximately 15 min to complete). Accompanied by one classroom teacher, one author (Masashi Soga) explained the purpose of the questionnaires and read aloud each question to the children to check whether they were able to understand its meaning. Classroom teachers also carefully observed the manner in which each child answered the questions. Children were not allowed to communicate with each other during the survey. If children had questions, they were allowed to ask for clarification. Each child received a three-page questionnaire written in Japanese. This asked about four topics: (1) frequency of direct and (2) vicarious experiences of nature, (3) affective attitudes toward biodiversity, and (4) willingness to conserve biodiversity.

### 2.2. Frequency of Direct Experience of Nature

To measure the frequency of direct experiences of nature, children were asked three questions about how frequently they participated in the following three types of nature-based activities on a daily basis. These were: (1) “How frequently do you visit neighborhood natural places (e.g., urban parks, woodlands, grasslands, riparian greenspace)?” (2) “How frequently do you touch or pick plants or flowers in neighborhood natural places?” and (3) “How frequently do you observe or touch wildlife (e.g., birds, insects) in neighborhood natural places?” These nature-based activities are the most common ones that children take part in outside school hours in this region. Picking plants or flowers in public places is not restricted by Japanese law. Responses were scored on a four-point scale (1 = never, 2 = seldom, 3 = sometimes, 4 = often); we explained to the children that these items roughly correspond to “less than once a month”, “almost every month”, “almost every week”, and “almost everyday”, respectively. Because these three types of nature-based activities are closely associated with each other, we used the mean of these three items as a measure of the frequency of direct experiences of nature. Internal consistency was acceptable (Cronbach alpha = 0.67).

### 2.3. Frequency of Vicarious Experience of Nature

To measure the frequency of vicarious experiences of nature, children were asked two questions. These were: (1) “How frequently do you read books or watch TV programs about nature or wildlife?” and (2) “How frequently do you talk about nature or wildlife with your parents or friends?” Responses were scored on a four-point scale (1 = never, 2 = seldom, 3 = sometimes, 4 = often). Responses to these two questions were used as separate measures of the frequency of vicarious experiences of nature.

### 2.4. Affective Attitudes toward and Willingness to Conserve Biodiversity

To measure children’s affective attitudes toward and willingness to conserve biodiversity, part of the questionnaire presented color photographs of 16 locally common animal species. In the present study, we only focused on animals to prevent confounding factors that can come from including plants: trees and flowers are different from animals in that they are normally static and not aggressive. We chose a diverse set of common animal species, with reference to the list of animal species in the study region. These species was comprised of lady beetle (*Coccinella septempunctata*), slug (*Meghimatium bilineatum*), earthworm (*Megascolecidae* spp.), sow bug (*Armadillidium vulgare*), butterfly (*Graphium sarpedon*), bat (*Pipistrellus abramus*), mantis (*Tenodera aridifolia*), cricket (*Teleogryllus emma*), cicada (*Hyalessa maculaticollis*), bee (*Xylocopa appendiculata circumvolans*), water strider (*Aquarius paludum paludum*), gecko (*Gekko japonicus*), bird (*Passer montanus*), dragonfly (*Orthetrum albistylum speciosum*), snake (*Elaphe climacophora*), and earwig (*Anisolabis maritima*). All of these are commonly seen in the study region and were referred to in the questionnaire by their common name at the species-level and, where appropriate, as “earthworm”, “bat” “gecko”, and “snake”, respectively. Although there are many other species that children can find locally, for the purpose of this study, we restricted our attention to the above species.

For each animal species, children were asked three questions, and responses were scored on a three-point scale. These were: (1) ‘‘Do you like this animal species?” (1 = like, 0 = no feeling, −1 = dislike); (2) “Would you like to get close to this animal species?” (1 = yes, 0 = no feeling, −1 = no); and (3) ‘‘Are you happy that this animal species is around you?” (1 = happy, 0 = no feeling, −1 = unhappy). The mean of these items (Cronbach alpha = 0.92) was used to form a measure of children’s affective attitudes toward biodiversity. Scores for affective attitudes toward biodiversity (hereafter attitude scores) ranged from −16 to 16. To measure children’s willingness to conserve biodiversity, they were also asked whether they would like to conserve each animal species (“Would you like to protect this animal species?”) (1 = yes, 0 = no feeling, −1 = no). Scores for willingness to conserve biodiversity (hereafter willingness scores) also ranged from −16 to 16.

For each child, attitude and willingness scores associated with each animal species were also calculated as well, by averaging the children’s attitudes and willingness scores (−1, 0, or 1) for each separately. Attitude and willingness scores for animal species both ranged from −1 to 1.

### 2.5. Statistical Analysis

We used path analysis to examine the relationships between the frequency of direct and vicarious experiences of nature, gender, and children’s affective attitudes toward (attitude scores) and willingness to conserve biodiversity (willingness scores) (Figure 1). In order to structure the models, we use the “lavaan” package and the “sem” function (ver. 0.5-18) [30] in R (ver. 3.2.1) [31]. In the model, the frequencies of direct and vicarious experiences of nature were used as explanatory variables. We also included gender as an explanatory variable to control for its potential confounding effects on children’s attitudes toward animal species. Gender was treated as a categorical variable: “female” was used as a reference category. Willingness scores were used as a response variable. Attitude scores were used as a mediating factor between the above explanatory and response variables, *i.e.*, a variable that transmits the indirect effects of explanatory variables (frequency of direct and vicarious experiences of nature and gender) on a response variable (willingness scores) (Figure 1). Since our preliminary analysis showed no significant effect of children’s age on attitudes toward biodiversity, we did not include it in the model. We performed a bootstrap analysis (based on 1000 replications) to calculate bias-corrected bootstrap 95% confidence intervals (CI) of coefficients for each path and mediation (indirect) effect in the model. We interpreted the significance of estimates for mediation effects by testing whether the 95% CI overlapped zero.

As the initial hypothetical model shown in Figure 1 yielded a good fit to the data, we interpreted the results based on that model. In the present study, we did not perform an additional “specification search” to find a model with good fit by trimming paths. This is because the final models chosen in this way capitalize on chance, and thus these models are less likely to extrapolate to other samples [32]. Overall fit of the models was determined by the root mean square error of approximation (RMSEA) and comparative fit index (CFI). Values for RMSEA and CFI range from 0.00 to 1.00, with values closer to 0.00 and 1.00 indicating a good model fit, respectively. Goodness of fit was assessed based on the following criteria: RMSEA < 0.05, CFI > 0.95.

As well as for the overall 16 animal species, path analysis was performed for different groups of species. We grouped the 16 animal species based on their attitude and willingness scores, using the hierarchical cluster technique with Ward’s linkage method. This uses a Euclidean distance metric to separate the set of animal species into groups (clusters). We used the “hclust” function implemented in R [31].

## 3. Results

### 3.1. Overall Results

**Figure 2:**
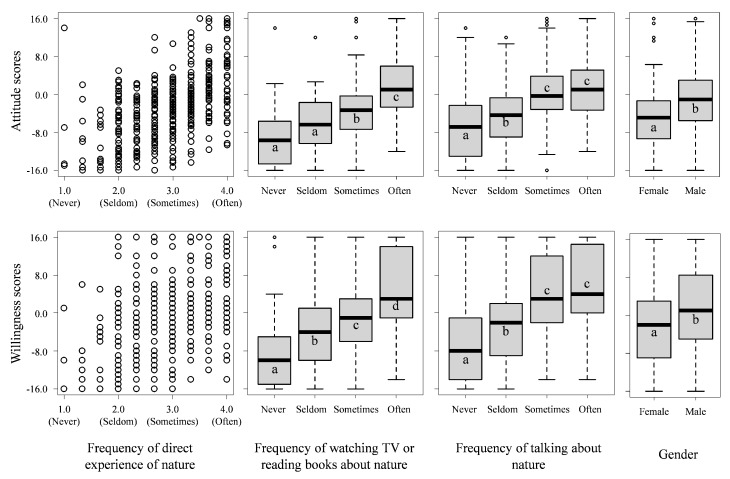
Relationships between the frequency of direct and vicarious experiences of nature, gender, and affective attitudes towards (attitude scores) and willingness to conserve biodiversity (willingness scores). The central bars in the box plot indicate the median, the ends of the boxes indicate the interquartile range, and the whiskers indicate the 10th and 90th quantiles. Different letters indicate significant differences between groups of data according to ANOVA with Tukey’s HSD test (see also Table S1).

Children’s attitude and willingness scores were positively associated with the frequency of both direct and vicarious experiences of nature (Figure 2). Children who had greater experience of nature showed higher attitude and willingness scores than those who had less (Figure 2, see the level of statistical significance by ANOVA with Tukey’s honestly significant difference (HSD) test in Table S1). Gender also had a significant effect on children’s attitude (ANOVA, df = 1, 395, *F* = 41.42, *p* < 0.001) and willingness scores (df = 1, 395, *F* = 13.96, *p* < 0.001): male children showed higher scores than female. Children’s attitude and willingness scores were positively correlated to each other (R = 0.69, *p* < 0.001).

### 3.2. Path Analysis

**Figure 3:**
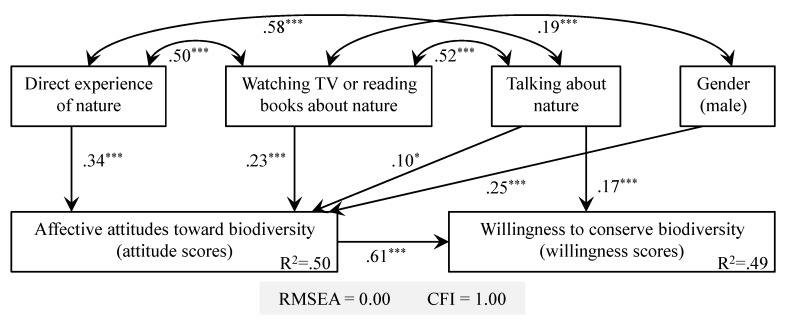
Model with standardized estimates of parameters (non-significant paths were omitted) predicting children’s attitudes toward (attitude scores) and willingness to conserve biodiversity (willingness scores). Asterisks indicate the significance levels (* *p* < 0.05; ** *p* < 0.01; *** *p* < 0.001).

The hypothesized model showed a good fit to the overall data for the 16 animal species (RMSEA = 0.00, CFI = 1.00) (Figure 3). The model showed that the frequency of direct and vicarious experiences of nature and gender had significant effects on attitude scores. The frequency of talking about nature was the sole variable that affected willingness scores directly (Figure 3). Effects of the frequency of direct and vicarious experiences of nature on willingness scores were both mediated by attitude scores (see Table S2).

### 3.3. Subgroup Analysis

**Figure 4:**
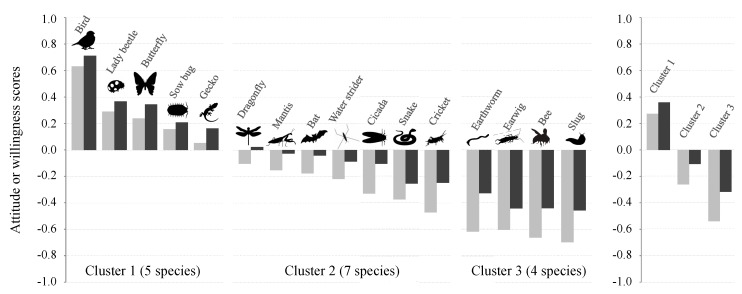
Attitude (**gray bars**) and willingness scores (**black bars**) for 16 animal species (**left panel**) and for three clusters (averaged values) (**right panel**). Species classification (Clusters 1, 2, and 3) were based on Ward’s dendrogram of a hierarchical cluster analysis (see also Figure S2).

There was marked variation in children’s attitude and willingness scores among the 16 animal species (Figure 4). With Ward’s hierarchical cluster analysis based on animal species’ attitude and willingness scores, 16 animal species were classified into three main groups: Cluster 1 (bird, lady beetle, butterfly, sow bug, and gecko), Cluster 2 (dragonfly, mantis, bat, water strider, cicada, snake, and cricket), and Cluster 3 (earthworm, earwig, bee, and slug) (Figure S2). Animal species’ attitude and willingness scores were highest for Cluster 1 and lowest for Cluster 3 (Figure 4), suggesting that Clusters 1 and 3 are the most attractive and unattractive species groups, respectively.

**Figure 5:**
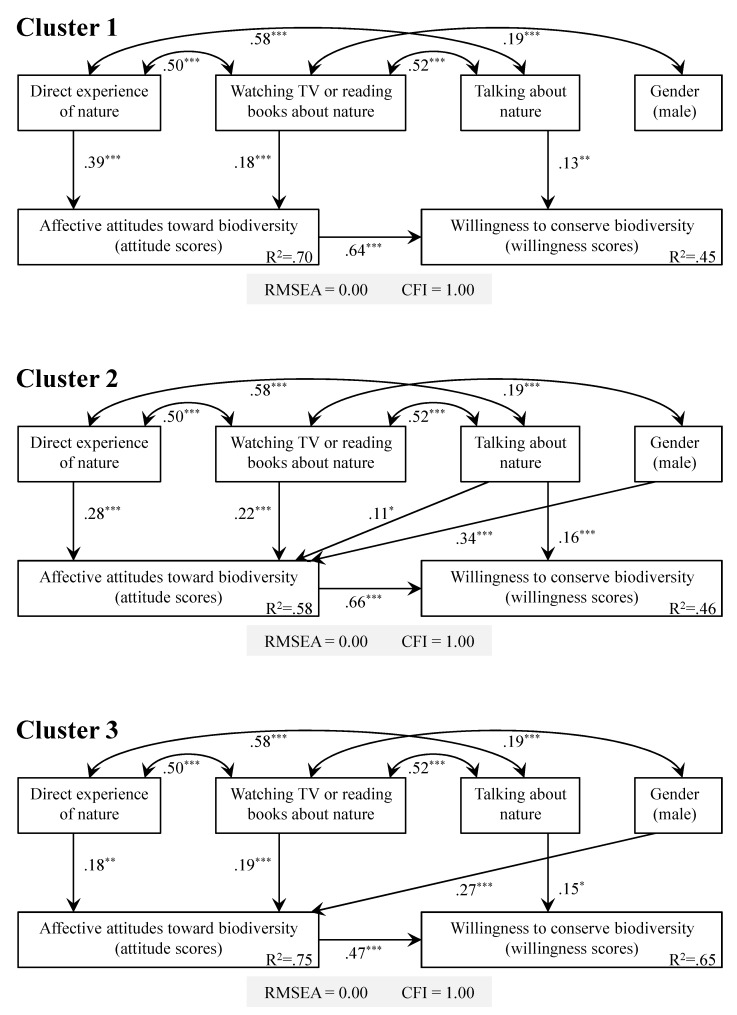
Models with standardized estimates of parameters (non-significant paths were omitted), predicting children’s attitudes toward (attitude scores) and willingness to conserve biodiversity (willingness scores) for species grouped into Cluster 1, 2, and 3 (see Figure 4). Asterisks indicate the significance levels (* *p* < 0.05; ** *p* < 0.01; *** *p* < 0.001).

The hypothesized model showed a good fit to the data of each cluster (RMSEA = 0.00, CFI = 1.00) (Figure 5). The overall patterns in the path analytic models appeared similar among the three clusters (Figure 5). For all clusters, effects of direct and vicarious experiences of nature on willingness scores were both mediated by attitude scores (see Table S2), and the frequency of talking about nature was the sole variable that affected willingness scores directly (Figure 5).

## 4. Discussion

This study is, to our knowledge, the first to evaluate the effects of “direct” and “vicarious” experiences of nature on children’s affective attitudes toward and willingness to conserve biodiversity. In our models, the frequency of direct experiences of nature was positively correlated to children’s affective attitudes toward local biodiversity (Figure 3). This result is in line with the well-documented hypothesis that children who frequently participate in nature-based activities are likely to develop greater emotional affinity to nature [17,18,19]. This study was conducted in Tokyo, one of the world’s largest mega-cities, and thus it suggests that, even in highly urbanized regions, neighborhood natural environments (e.g., woodlands, parks, riparian greenspace) act as places where children can be connected to nature, both physically and emotionally. Given that neighborhood greenspace (e.g., urban parks) is the main and perhaps the only area where children can contact with nature daily, preserving or restoring natural environments close to homes may be critically important not only for conserving urban biodiversity [33] but also for reducing the extinction of experience for urban dwellers [34,35]. To quote Pyle [13], “[w]e must save not only the wilderness but the vacant lots, the ditches as well as the canyonlands, and the woodlots along with the old growth”.

Children’s affective attitudes toward nature should not be determined simply by the frequency of direct contact with nature. Indeed, previous studies exploring effects of different types of nature experiences on children’s environmental attitudes and behavior have suggested a significant positive impact of vicarious nature experiences (e.g., talking about the environment, watching nature films, and reading books about the environment) [22,36,37]. In the present study, the frequency of vicarious experiences of nature, as well as that of direct experiences, was positively associated with children’s affective attitudes toward local biodiversity (Figure 3). This result suggests that, in circumstances where a given amount of natural environment cannot immediately be preserved within the home ranges of children, vicarious experiences (e.g., photographs or videos representing nature or wildlife) could, to some extent, be used as a subsidiary tool in attracting their interest in and care for biodiversity.

According to the biophilia hypothesis, humans have an innate tendency to affiliate with living organisms, both positively (biophilia) and negatively (biophobia) [38,39]. Indeed, we observed marked variation in children’s preferences among the 16 animal species: some animal species (e.g., bird, lady beetle, and butterfly) attracted favorable attention but others did not (e.g., earthworm, slug, and bee) (Figure 4). This result corroborates previous work demonstrating the high popularity of certain animal species over others; it has been reported that people show positive feelings toward birds and butterflies [19,25,28,40], whereas they tend to dislike or fear small dull-colored invertebrates, bats, and snakes [28,41,42]. Nevertheless, the reported positive relationship between the frequency of nature experiences and children’s attitude scores was evident both for attractive and unattractive species groups (Figure 5). This result is important because it suggests that interacting with nature not only increases children’s positive feelings toward animals (biophilia) but also alleviates negative feelings (biophobia) [19]. That said, our study does not fully cover Wilson’s concept of biophilia [38,39]. Indeed, biophilia means a psychological orientation of being attracted to all living organisms and processes, which does not give priority to animals. Hence, future research should extend the current study to different fauna and flora, natural settings, and ecosystems, which might offer us more comprehensive results.

In this study, female children were more likely to show negative responses to animal species than male (Figure 2), which is consistent with previous research showing significant gender differences in attitudes toward animals [19,28]. Although it is not easy to identify a mechanism to explain this finding, previous work suggests that women’s elevated fear for animals, such as spiders and snakes, is an intrinsic property [43]. Indeed, in our study, gender differences in children’s attitude scores were particularly evident for unattractive species groups (Clusters 2 and 3) (Figure 5). Another possible reason for this result is that male children are more likely to face social pressures to be unafraid of animals, which may, in turn, prevent them from manifesting negative responses [19].

We observed a strong correlation between children’s attitude and willingness scores. Path analysis also revealed that children’s affective attitudes toward biodiversity acted as a mediating factor between experience of nature and willingness to conserve biodiversity. In other words, affective attitudes transmitted indirect effects of frequencies of experiences of nature on willingness to conserve biodiversity. These results highlight that individuals’ affective attitude toward nature is a key driver of their motivation to protect it [24,25,26,27,44]. Similar to our study, Zhang *et al.* [19] reported that participation in nature-based activities increased children’s positive attitudes toward wildlife, which enhanced their willingness to conserve it and show pro-environmental behavior. In our models, although most explanatory variables influenced children’s willingness to conserve biodiversity indirectly, the frequency of talking about nature with parents or friends had a direct effect (Figure 3). One plausible reason for this result is that children’s positive beliefs and thoughts toward local biodiversity conservation were likely encouraged by conversations with parents or friends about nature and wildlife. Indeed, recent studies suggest a link between family members’ values toward the environment and children’s environmental attitudes and behavior [17,45].

Although we obtained clear results, inevitably this study has its limitations. First, we only studied one elementary school in Tokyo, and thus our results could be affected by the specific cultural context. Indeed, Japan has a traditional culture with an appreciation for nature, especially for insects [46]. Thus, caution is warranted in any generalization of the findings. Second, because our data were collected through self-reported questionnaires completed by the children, they may suffer from a recall bias. Future research could use more objective measures of frequency, duration, and intensity of experience with nature. Third, this study employed a cross-sectional design, and thus we were not able to establish the bidirectional relationships among variables. Further longitudinal studies could clarify this issue. Fourth, the findings of this analysis should be interpreted with some caution, due to some degree of multicollinearity between direct and vicarious experiences. Fifth, our data could not identify the specific type and frequency of nature experiences that may have a key role in developing children’s positive attitudes toward nature, although such information is critical for policy making. Finally, and this is the nature of attitudinal studies, children’s attitudes toward biodiversity do not necessarily lead to actual conservation behavior (see [47]). Hence, future research ought to investigate how the loss of contact with nature affects people’s participation in pro-environmental activities more directly.

## 5. Conclusions

In conclusion, our study has two important implications. On the one hand, it highlights that the ongoing disconnection of children from nature (especially neighborhood natural environments and biodiversity) can, from a long-term viewpoint, have serious consequences for future support for biodiversity conservation [6]. On the other hand, it provides a hopeful message that, even in our rapidly urbanizing world, there are still many opportunities to reconnect children with nature [3], which is not limited to direct experiences of nature but also vicarious ones. We therefore suggest that children should be encouraged to experience nature and be provided with various types of these experiences. As today’s children are the environmental stewards of the future, redressing the extinction of experience of nature would be the fundamental step toward building a sustainable society.

## Supplementary Materials

The following are available online at www.mdpi.com/1660-4601/13/6/529/s1, Figure S1: Land use map of the region around the study school (black square). In the middle of the map is the Tama River, the biggest river in Tokyo. Data from the Ministry of Land Infrastructure and Transport (http://www.mlit.go.jp/crd/daisei_ryokuhi_index.html), Figure S2: Ward’s dendrogram of a hierarchical cluster analysis conducted on 16 animal species, Table S1: Summary of Tukey’s HSD test (see Figure 2 in the main text), Table S2: Unstandardized and standardized parameter estimates for mediated relationships in the model (see Figure 3 and Figure 5 in the main text).
