# Supplementary material for: Both Direct and Vicarious Experiences of Nature Affect Children’s Willingness to Conserve Biodiversity
Source: Int J Environ Res Public Health. 2016 May 25;13(6):529. doi: 10.3390/ijerph13060529 (PMC4923986; doi:10.3390/ijerph13060529)
Supplement: Supplementary file 1 [file ijerph-13-00529-s001.pdf]

# Both Direct and Vicarious Experiences of Nature Affect Children's Willingness to Conserve Biodiversity

Masashi Soga, Kevin J. Gaston, Yuichi Yamaura, Kiyo Kurisu and Keisuke Hanaki

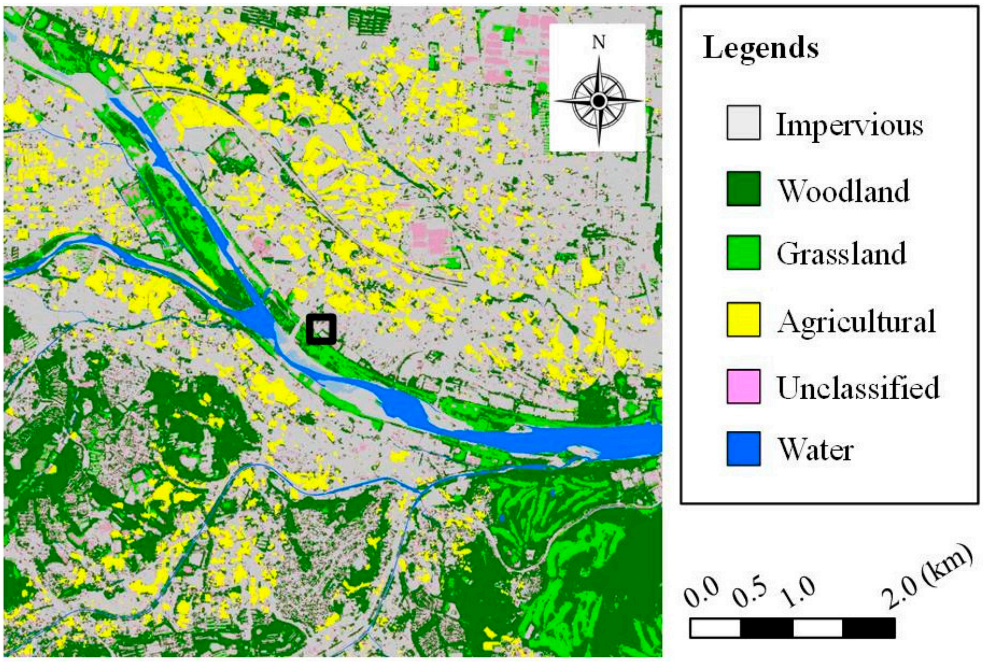

Figure S1. Land use map of the region around the study school (**black square**). In the middle of the map is the Tama River, the biggest river in Tokyo.

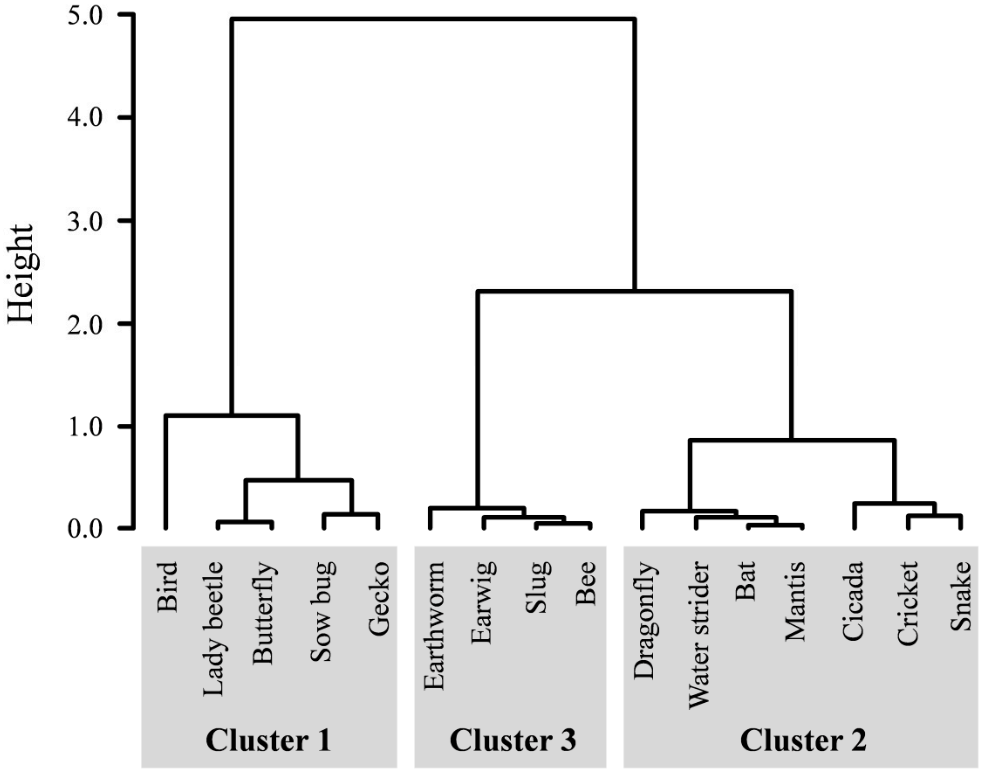

Figure S2. Ward's dendrogram of a hierarchical cluster analysis conducted on 16 animal species.

Table S1. Summary of Tukey's HSD (honestly significant difference) test (see Figure 2 in the main text).

Combination	*t* Value	*p* Value
Watching TV or reading books about nature		
Attitude scores		
Never *vs.* Seldom	2.47	0.07
Never *vs.* Sometimes	4.85	<0.001
Never *vs.* Often	9.76	<0.001
Seldom *vs.* Sometimes	3.04	0.01
Seldom *vs.* Often	9.64	<0.001
Sometimes *vs.* Often	7.80	<0.001
Willingness scores		
Never *vs.* Seldom	2.88	0.02
Never *vs.* Sometimes	5.14	<0.001
Never *vs.* Often	8.42	<0.001
Seldom *vs.* Sometimes	2.83	0.02
Seldom *vs.* Often	7.28	<0.001
Sometimes *vs.* Often	5.32	<0.001
Talking about nature		
Attitude scores		
Never *vs.* Seldom	2.99	0.02
Never *vs.* Sometimes	8.16	<0.001
Never *vs.* Often	6.93	<0.001
Seldom *vs.* Sometimes	6.09	<0.001
Seldom *vs.* Often	5.13	<0.001
Sometimes *vs.* Often	0.67	0.91
Willingness scores		
Never *vs.* Seldom	3.77	0.001
Never *vs.* Sometimes	8.80	<0.001
Never *vs.* Often	7.47	<0.001
Seldom *vs.* Sometimes	5.99	<0.001
Seldom *vs.* Often	5.12	<0.001
Sometimes *vs.* Often	0.72	0.89

Table S2. Unstandardized and standardized parameter estimates for mediated relationships in the model (see Figures 3 and 5 in the main text).

Path	Estimate	95% CI		Standardized Estimate
		Lower	Upper	
**All species (16 species)**				
Direct experience → Affective attitudes → Willingness to conserve	2.60	1.54	3.75	0.20
Reading and watching → Affective attitudes → Willingness to conserve	1.40	0.78	2.08	0.14
Talking → Affective attitudes → Willingness to conserve	0.62	0.05	1.14	0.06
Gender → Affective attitudes → Willingness to conserve	2.76	1.82	3.77	0.15
**Cluster 1 (5 species)**				
Direct experience → Affective attitudes → Willingness to conserve	0.99	0.64	1.36	0.25
Reading and watching → Affective attitudes → Willingness to conserve	0.36	0.15	0.58	0.12
Talking → Affective attitudes → Willingness to conserve	0.11	−0.10	0.31	0.04
Gender → Affective attitudes → Willingness to conserve	−0.04	−0.38	0.24	−0.01
**Cluster 2 (7 species)**				
Direct experience → Affective attitudes → Willingness to conserve	1.22	0.70	1.73	0.19
Reading and watching → Affective attitudes → Willingness to conserve	0.75	0.43	1.10	0.15
Talking → Affective attitudes → Willingness to conserve	0.37	0.04	0.69	0.07
Gender → Affective attitudes → Willingness to conserve	2.11	1.62	2.76	0.22
**Cluster 3 (4 species)**				
Direct experience → Affective attitudes → Willingness to conserve	0.32	0.11	0.52	0.09
Reading and watching → Affective attitudes → Willingness to conserve	0.26	0.12	0.41	0.09
Talking → Affective attitudes → Willingness to conserve	0.13	−0.01	0.28	0.05
Gender → Affective attitudes → Willingness to conserve	0.69	0.43	0.98	0.13

© 2016 by the authors; licensee MDPI, Basel, Switzerland. This article is an open access article distributed under the terms and conditions of the Creative Commons by Attribution (CC-BY) license (http://creativecommons.org/licenses/by/4.0/).
